# Genomic signatures of rapid adaptive divergence in a tropical montane species

**DOI:** 10.1098/rsbl.2021.0089

**Published:** 2021-07-28

**Authors:** Per G. P. Ericson, Martin Irestedt, Huishang She, Yanhua Qu

**Affiliations:** ^1^ Department of Bioinformatics and Genetics, Swedish Museum of Natural History, PO Box 50007, 10405, Stockholm, Sweden; ^2^ Key Laboratory of Zoological Systematics and Evolution, Institute of Zoology, Chinese Academy of Sciences, Beijing 100101, People's Republic of China

**Keywords:** bowerbirds, divergent adaptation, ‘sky island’ diversification

## Abstract

Mountain regions contain extraordinary biodiversity. The environmental heterogeneity and glacial cycles often accelerate speciation and adaptation of montane species, but how these processes influence the genomic differentiation of these species is largely unknown. Using a novel chromosome-level genome and population genomic comparisons, we study allopatric divergence and selection in an iconic bird living in a tropical mountain region in New Guinea, Archbold's bowerbird (*Amblyornis papuensis*). Our results show that the two populations inhabiting the eastern and western Central Range became isolated *ca* 11 800 years ago, probably because the suitable habitats for this cold-tolerating bird decreased when the climate got warmer. Our genomic scans detect that genes in highly divergent genomic regions are over-represented in developmental processes, which is probably associated with the observed differences in body size between the populations. Overall, our results suggest that environmental differences between the eastern and western Central Range probably drive adaptive divergence between them.

## 1. Introduction

Mountain regions are among the biologically richest areas in the world and of great importance for conservation [1,2]. This extraordinary biodiversity is even more accentuated in tropical mountains where long-term climatic changes interacting with the heterogeneous topography provide multiple opportunities for speciation and adaptation [3,4]. Consequently, tropical mountains house a larger number of restricted-range, ecological specialists than do temperate mountainous regions [4,5]. During the Pleistocene, glacial cycles further accelerated the diversification of mountain species as the climate changes pushed habitats and their inhabitants up and down along elevational gradients [6]. In warmer periods, species may have been split into several ‘sky island’ populations [7,8] as they became isolated at mountaintops surrounded by unfavourable habitats. Understanding how ‘sky island’ diversifications influence the genomic landscape and divergent adaptations is important for determining future conservation measures in these species, not least at a time when the global loss of biological diversity is accelerating (e.g. [9]).

Herein we address genomic divergence and adaptation in Archbold's bowerbird *Amblyornis papuensis*, Ptilonorhynchidae (nomenclature follows [10]), supposedly one of the rarest birds in New Guinea [11]. It inhabits frost-prone high mountains at elevations between 2300 and 3660 m, higher than most birds in New Guinea [12–14]. It is resident and divided into two geographically isolated and sedentary populations (*sanfordi* in the eastern Central Range and *papuensis* in the western Central Range) that differ in size and plumage colour [14]. Constituting a classic ‘sky island’ species, *A. papuensis* provides unique opportunities to study the genomic signature of local adaptations in allopatric populations. In the study, we generated a nearly chromosome-level genome of a closely related species, southern white-eared catbird *Ailuroedus stonii*, and studied comparative population genomics of *A. papuensis*. We expect to find that the two populations have diverged during a warm interglacial period and subsequently decreased their population sizes when they retreated to higher elevation. We hypothesize that the populations have evolved genomic adaptations in response to the different environmental pressures in their respective ‘sky island’ region. Our study provides novel insights into how the Late Pleistocene climate and environmental heterogeneity of tropical mountains contributed to the rapid adaptive divergence between ‘sky island’ populations.

## 2. Material and methods

### (a) De novo genome and resequencing data generation

We generated a nearly chromosome-level genome of the closely related *A. stonii* (see the electronic supplementary material for specimen details). We sequenced paired-end (180 bp), mate-pair (3 and 5–8 kb) and 10× genomics chromium libraries, using Illumina HiSeq X by Science for Life Laboratory (National Genomics Institute, Stockholm). We assembled the genome using ALLPATHS_LG [15] and further improved it by Hi-C sequencing and using the HiRise assembly pipeline (Dovetail Genomics [16]). In total, we obtained 218 Gb data. We extracted DNA from toepads of eight taxonomically well-identified and vouchered museum study skins of *A. papuensis* collected between 1938 and 1961 with Illumina NovaSeq 6000 (electronic supplementary material, table S1). We deleted 5 bp from both ends of cleaned reads to reduce ‘noise’ caused by DNA degradation (a standard procedure for museum specimens [17]). We used BWA mem v. 0.7.12 [18] to map the clean reads against the 23 largest scaffolds, which cover 97% of the *A. stonii* genome. We called single nucleotide polymorphisms (SNPs) using *mpileup* in Samtools v. 1.4 [19], applying a minimum genotype quality of 10.

### (b) Population structure

Phylogenetic relationships were inferred by analysing 80 157 randomly drawn SNPs with SNAPP v. 1.3.0 in BEAST2 v. 2.4.8 [20,21]. We chose uninformative distributions as priors, sampled mutation rate from an inverse gamma distribution, used a Yule prior for species tree, and set the lambda parameter to a uniform distribution (0–1). We ran the analysis for 2 000 000 iterations (the first 200 000 was discarded as burn-in). Convergence of the Markov chain Monte Carlo chains was assessed by checking that effective sample size values exceeded 200. We plot trees in the posterior sample using DensiTree v. 2.1.11 [22]. We estimated genetic population structure with principal component analysis (PCA) using *smartpca* in Eigensoft v. 6.1.4 [23].

### (c) Demographic history

To estimate historical demography, we applied Fastsimcoal v. 2.6 [24] to a two-dimensional, unfolded site frequency spectrum generated from a 76 Mb genomic region containing none of the 5% most highly divergent windows. We kept monomorphic sites and used the reference genome to polarize ancestral states. Four demographic models were tested: no decrease of population sizes after they split and no gene flow between them (M1); no decrease of population sizes but gene flow occurring (M2); changes of population sizes after they split but no gene flow (M3); changes of population sizes and gene flow occurring (M4) (electronic supplementary material, figure S1).

Akaike information criterion (AIC) [25] was used to select which model fit observed data best. For the best-fit model, we ran 1000 replicates, each including 20 estimation loops with 200 000 coalescent simulations. We calculated statistics for demographic parameters based on the 5% most likely runs.

We used PopSizeABC [26] to estimate temporal fluctuations in population size. We set the recombination rate to 1.0 × 10^−8^, mutation rate to 4.6 × 10^−9^ per generation and a generation time of 5.35 years [27–29]. Summary statistics of the allele frequency spectrum and linkage disequilibrium were calculated at 21 discrete time windows (2400–130 000 years) based on the empirical dataset and then compared with the corresponding statistics calculated from 400 000 simulated datasets.

### (d) Environmental heterogeneity analysis

We tested environmental heterogeneity using 19 bioclimatic variables obtained for 1939 and 2938 randomly sampled sites at 2600–2800 m.a.s.l. within the core-distributions (as determined from [12–14]) of the western and eastern populations in WorldClim v. 2.1 database [30] (see the electronic supplementary material). We performed PCA on the Z-transformed dataset in R (*prcomp*) and used two-tailed *t*-tests to test for significance in both the 19 bioclimatic variables and the principal components.

### (e) Selection analysis

The landscape of genomic divergence between the two populations was estimated by calculating *f*_st_ and *D*_xy_ values in non-overlapping 50 kb windows (estimations using PopLDdecay [31] showed linkage disequilibrium to decay within this window size). We identified highly divergent genomic regions using Z-transformed *f*_st_. To determine the cutoff value, we generated 2000 genome-wide nominal values through simulations under the inferred demographic history (M4). We used the top 5% percentile value of the simulated distributions as cutoff. The chicken gene set (Gallus.gallus.5.0.cds) in BLAST+ v. 2.6.0 [32] was used to annotate these regions and the identified genes subjected to enrichment analysis using Panther [33]. We also searched for evidence of selective sweeps in each population using SweeD [34]. To test if the observed pattern of divergent selection is driven by stochastic processes, we ran permutation tests by generating 10 000 random samples with the same numbers of genes as observed in the *F*_ST_, *D*_XY_ and SweeD analyses, respectively. For each generated sample we annotated the genes for their relevant functions in gene ontology (GO) to obtain null distributions for the proportion of development genes given a certain total number of genes. We compared the observed number of genes to the null distributions and regarded observed values larger than the 95th percentile of the null distribution to be statistically significant.

## 3. Results and discussion

The novel *A. stonii* genome has a size of 1092 Mb and consists of 2364 scaffolds (N50 scaffold size is 75 Mb and N50 contig size is 436 kb). The 23 largest scaffolds cover 97% of the genome, and 22 of these match chicken chromosomes 1–20 (electronic supplementary material, table S2). We used these scaffolds for downstream analyses and refer to them using chicken chromosome numbers.

**Figure 1.**
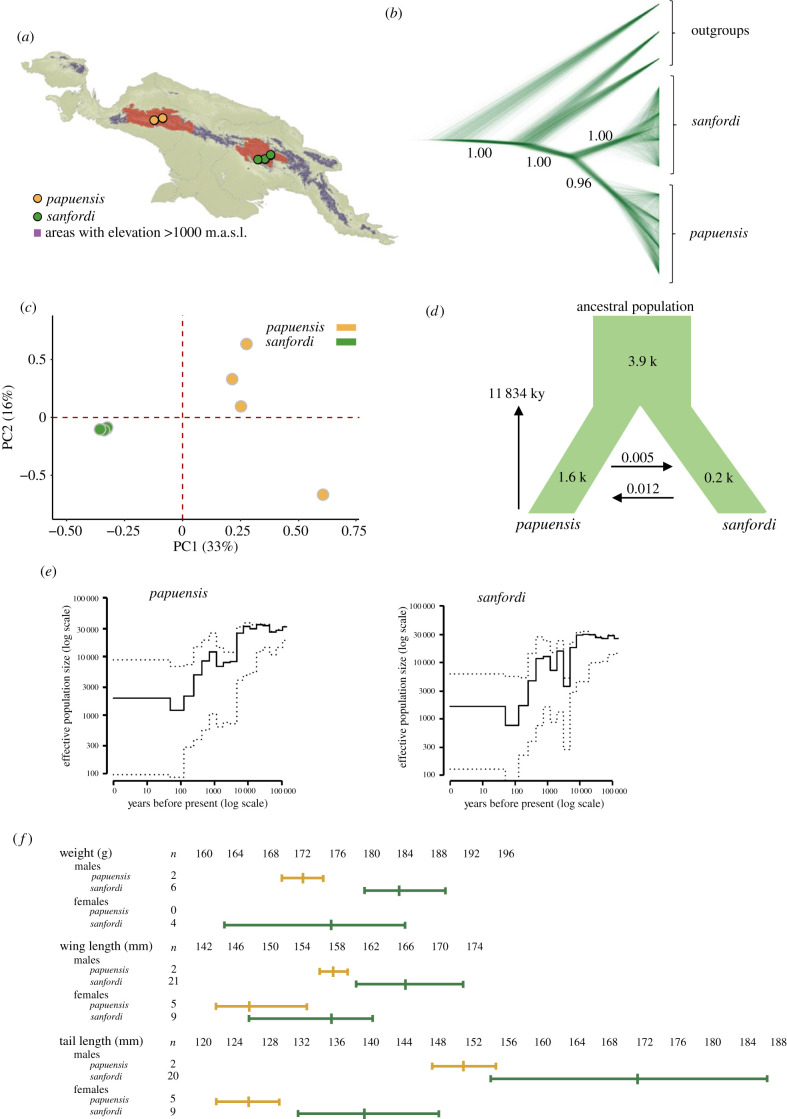
(*a*) *Amblyornis papuensis* occupies high mountainous mossy beech (*Nothofagus*) forests in the western (*papuensis*) and eastern (*sanfordi*) parts of the Central Range in New Guinea (reddish shade, from [14]). Coloured dots represent sampling sites and purple colour elevations greater than 1000 m.a.s.l. (*b*) Phylogenetic relationship within *Amblyornis papuensis* inferred by SNAPP (based on 80 157 randomly sampled SNPs). *Amblyornis macgregoriae*, *Amblyornis subalaris* and *Amblyornis inornata* serve as outgroups. Bootstrap values greater than 0.95 are indicated. (*c*) Principal component analysis of 2 467 355 SNPs. (*d*) Estimates of demographic parameters based on the best-fit model (M4) inferred with Fastsimcoal. (*e*) Temporal variation in effective size (*N*_e_) inferred by PopSizeABC. (*f*) Size comparisons of adult individuals of the western (*papuensis*) and eastern (*sanfordi*) populations (from [27], see also the electronic supplementary material, table S7).

The SNAPP result showed that the eight individuals of *A. papuensis* fall into two clades, each receiving 100% support, corresponding to the two recognized subspecies *papuensis* and *sanfordi* (figure 1*a*,*b*). The PCA analysis also showed a clear separation between the two populations in the first two PCs (figure 1*c*; electronic supplementary material, figure S2). Although the individual span in nucleotide diversity is larger among the individuals of *papuensis* than *sanfordi*, the average nucleotide diversity is similar in the two populations (0.08% in *papuensis* and 0.09% in *sanfordi*) as is the average standardized multilocus heterozygosity (1.01 in *papuensis* and 1.00 in *sanfordi*).

The AIC comparison supports model M4, decreasing population sizes with gene flow as the best-fit (figure 1*d*; electronic supplementary material, table S3). Based on this we estimated that the two populations split around 11.8 kya (95% confidence interval (CI): 10.2–12.4 kya) with a negligible gene flow (0.005 individuals per generation from *papuensis* to *sanfordi* and 0.012 in the opposite direction, figure 1*d*; electronic supplementary material, table S4). After their split, the populations underwent an almost 10-fold decrease of their effective sizes (figure 1*e*).

The preferred habitats of *A. papuensis* are montane forests dominated by *Nothofagus* trees [14]. Today these forests are found between 1800 and 3000 m.a.s.l. [35], similar to the elevational distribution of *A. papuensis* [14]. During glaciations, glaciers covered several mountaintops and pushed the *Nothofagus* forests downwards to 800–1800 m.a.s.l. [36,37]. This expanded the area of habitats suitable for *A. papuensis*, allowing an increase of its population size. The gradually warmer Late Pleistocene climate pushed the cold-adapted *Nothofagus* forests to higher elevations. The split between the two *A. papuensis* populations is estimated to about 11.8 kya and is probably a consequence of the steady decrease of suitable habitats.

**Figure 2.**
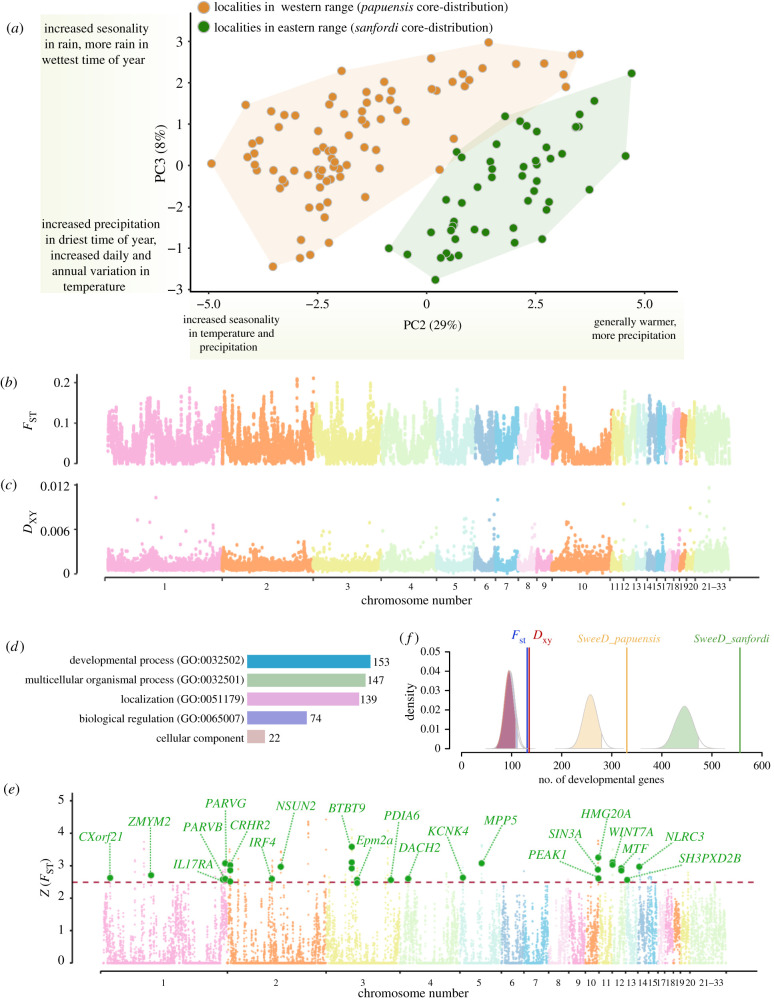
(*a*) PCA of 19 bioclimatic variables observed at localities within core-distributions of *papuensis* and *sanfordi* (for component loadings see the electronic supplementary material, table S6). (*b*) and (*c*) Genome-wide variation in *F*_st_ and *D*_xy_. (*d*) GO enrichment results from the genes identified within the top 5% highly divergent genomic regions between the populations. (*e*) Genome-wide distribution of *Z*(*F*_st_) in 50 kb windows. Genes linked to developmental GO terms are indicated (the dotted line indicates the *Z*(*F*_st_) value for windows showing top 1% differential selection between the populations). (*f*) Permutation tests to explore the probability of whether stochastic processes alone can explain the numbers of genes related to development observed in analyses of divergent selection between *papuensis* and *sanfordi* (*Z*(*F*_st_) and *D*_xy_) and of selective sweeps within each of them. Null distributions are shown with the 95th percentiles shaded. Vertical lines show observed values.

We observed significant differences between the core-distributions of *papuensis* and *sanfordi* in 10 out of 19 bioclimatic variables (electronic supplementary material, tables S5 and S6). When plotting PC2 and PC3 (proxies for temperature and precipitation), the localities in the eastern and western Central Range form two almost non-overlapping clusters (figure 2*a*). The loading scores also differ significantly between these areas (two-tailed *t*-test, PC2: *t*_249_= 34.8, *p* < 0.001; PC3: *t*_249_ = 10.2, *p* < 0.001). Although seasonality in both temperature and precipitation show the largest differences together with the annual precipitation (electronic supplementary material, table S5), the combined effect of the bioclimatic variables is a relatively colder and more humid climate in the eastern Central Range, where *sanfordi* lives. Adaptation to local environmental conditions often explains intraspecific size variation in birds [38]. The two populations of *A. papuensis* exhibit considerable phenotypic differences, with *sanfordi* being generally larger than *papuensis*, particularly in the tail and wing lengths (figure 1*f*; electronic supplementary material, table S7) [27]. It is plausible that the observed phenotypic differences relate to variation in local climate (electronic supplementary material, table S5).

Given a recent split time, we found a high level of genetic divergence between the two populations, indicated by an average *F*_ST_ value of 0.0523 (95% CI: 0.0518–0.0528) and *D*_XY_ of 0.00123 (95% CI: 0.00122–0.00124), respectively (cf. figure 2*b*,*c*). Considering the negligible gene flow between the two populations, this divergence may have arisen by a strong genetic drift owing to isolation in different sky-island regions and drastically decreasing population sizes.

Notably, we found that the top 5% highly divergent windows (with *Z*(*F*_ST_) > 1.76), contain a large number of genes involved in developmental processes (153 of 493 genes, 31%). This proportion is significantly larger than expected from the genome background (3568 of 17 770 genes, Fisher's test *p* = 9.09 × 10^−6^). Our permutation tests further showed that this proportion of developmental genes is larger than expected by random chance alone (figure 2*f*). Consistently, the GO enrichment analysis of the 493 genes shows that 33% of the significantly over-represented GO terms are related to development processes (figure 2*d,e*; electronic supplementary material, table S8). A similar over-representation of developmental genes as for *Z*(*F*_ST_) is also observed in the *D*_XY_ analysis (electronic supplementary material, table S9). In addition, we found that the selective sweeps are stronger in *sanfordi* than in *papuensis*, shown by a larger average composite likelihood-ratio (CLR, 0.11 versus 0.04, Wilcoxon, *p* < 0.0001) as well as a larger number of highly selected regions (4104 versus 2184, *χ*^2^ 607.00, *p* < 0.0001, electronic supplementary material, figures S3 and S4). A functional annotation of these highly selected regions reveals a larger proportion of development genes than would be expected by random (figure 2*f*). Although our results show that divergent selection of the two populations has especially targeted developmental genes, we also observe other signals of selection in, e.g. biological regulation and multicellular organismal processes. The total genomic divergence is surely attributable to multiple forces, including interspecific competition, trophic niche utilization, sexual selection, etc. However, as developmental genes take the largest proportion of selective genes, we conclude that adaptive divergence in the developmental genes is the major component shaping the genomic landscape of divergence between the two populations. Previous studies of high-elevation animals have shown that changes in body size is linked to increased selection in developmental genes, e.g. in yak [39], ground tit [40], Tibetan chicken [41] and Eurasian tree sparrow [42]. We thus postulate that the phenotypic and genomic differences observed between the two populations of *A. papuensis* are likely to be either causal or indirect consequences of their adaptation to high-elevation environments. Based on these differences, as well as their geographical isolation, we suggest treating these populations as separate species, *A. papuensis* (Rand, 1940) and *A. sanfordi* (Mayr & Gilliard, 1950). This could help when determining future conservation policies for these very rare New Guinean birds.

## Data accessibility

Raw Illumina sequences are deposited in Sequence Reads Archive, National Center for Biotechnology Information, SRA accession no. PRJNA601961. The *A. stonii* genome assembly and data related to the analyses are deposited in Dryad Digital Repository: https://doi.org/10.5061/dryad.37pvmcvjr [43].
